# Super-Resolution Contrast-Enhanced Ultrasound Examination Down to the Microvasculature Enables Quantitative Analysis of Liver Lesions: First Results

**DOI:** 10.3390/life15070991

**Published:** 2025-06-20

**Authors:** Ulrich Kaiser, Ursula Vehling-Kaiser, Fabian Kück, Mia Gilanschah, Friedrich Jung, Ernst Michael Jung

**Affiliations:** 1 Medical Clinic and Polyclinic for Internal Medicine III, University Hospital Regensburg, 93053 Regensburg, Germany; 2 MVZ Dr. Vehling-Kaiser GmbH, 84036 Landshut, Germany; 3 Department of Medical Statistics, University Medical Center Göttingen, 37073 Göttingen, Germany; 4 Institute for Diagnostic Radiology and Interdisciplinary Ultrasound Center, University Hospital Regensburg, 93053 Regensburg, Germany; 5 Institute of Biotechnology, Molecular Cell Biology, Brandenburg University of Technology, 01968 Senftenberg, Germany

**Keywords:** contrast agent, ultrasonography, liver, intervention, microvascularization

## Abstract

This article investigates the first use of dynamic microvascularization of liver lesions in clinical evaluation using real-time super-resolution contrast-enhanced ultrasound (SR CEUS). A retrospective analysis of SR CEUS examinations of liver lesions was performed. All examinations were conducted using an SC7-1U convex probe after the bolus injection of the ultrasound contrast agent. Digital cine loops were stored for independent evaluation. The evaluation was performed with respect to parallel measuring lines (the diameter corresponded to the capillary density) in the area of the reference lesion, liver tissue, and liver capsule using statistical analysis. In total, 65 patients (female *n* = 28; male *n* = 37; average age 57.8 ± 17.2 years) were evaluated. The examined liver lesions were mostly benign masses (*n* = 46). Mild liver fibrosis (stage F1) was present in most cases (*n* = 35). The lesions examined were located at an average tissue depth of 6.07 ± 2.47 cm. The highest number of lesion measurements was observed in the malignant lesion group. Significant differences in the measurements were found when comparing benign lesions with the capillary region (*p* < 0.001) and normal liver tissue (*p* < 0.01). The use of SR CEUS opens up new possibilities for the quantification of neovascularization, assessment of microvascular changes, and evaluation of the follow-up of intrahepatic interventions.

## 1. Introduction

Medical ultrasound technology and applications have evolved over the past few years, and they continue to be improved or even newly developed [1]. Examples include various modern flow techniques, elastography measurements, and contrast agent methods [2,3,4]. As a result, medical questions can be answered with increasing accuracy, and a wider range of therapeutic applications can be used [5,6]. The detection and characterization of liver lesions is one of the most common reasons for the use of contrast-enhanced ultrasound (CEUS) [7,8,9,10,11]. According to the results of multicenter evaluations, recording wash-in and wash-out kinetics and assessing the vascularization pattern of solid liver lesions or complicated cystic changes are crucial to ensure diagnostic certainty [9,11].

In addition to assessing the severity of conspicuous lesions and space-occupying masses, modern CEUS can also be used to monitor treatment, such as systemic therapies, surgery, and ablative procedures.

Malignant liver lesions on CEUS are typically characterized by an irregular vascular pattern, rapid inhomogeneous contrast agent enhancement, and wash-out, beginning in the portal venous phase (after 60 to 90 s) and continuing into the late phase (5 to 7 min) [9,12]. For example, a distinction is made between a chaotic vascular pattern, as seen in hepatocellular carcinoma (HCC), and irregular, shunt-like, inhomogeneous hypercontrasting in the peripheral area in metastases, which appears in a ring-shape with subsequent wash-out phenomena increasing in the late phase. In assessing benign liver lesions, it is crucial to determine whether a regular vascular pattern is visible and whether it shows persistent enhancement without significant wash-out. In accordance with the guidelines, three forms of regular microvascularization for CEUS in benign lesions are described. For example, focal nodular hyperplasia (FNH) typically presents as a wheel-spoke pattern with arterial contrast from the center to the edge, whereas adenoma presents with arterial contrast from the edge to the center and a regular vascular pattern [12]. Hemangiomas have a nodular accumulation pattern that increases in size from the edge. In addition to assessing the seriousness of the lesion, CEUS is also very important in monitoring treatment after systemic therapies, local ablative procedures, or surgery, as the use of second-generation contrast agents means that CEUS no longer poses a relevant risk in cases of renal insufficiency and thyroid disorders [12,13,14,15]. The seriousness of the tumor disease was determined based on the size of the lesion, standardized using the RECIST criteria, and the extent of neovascularization as well as the distribution pattern categorized as “localized” or “generally diffuse.” Both categories can be evaluated very well using CEUS [7].

Therefore, using CEUS, therapies for liver tumors can be monitored with a high degree of diagnostic certainty. The key to successful tumor therapy is the complete reduction in the previous malignant tumor neovascularization. This should result in cyst-like areas of necrosis following resection or ablation and significant devascularization following chemotherapy or transarterial embolization (TACE).

When establishing new CEUS techniques, it is important to investigate to what extent the diagnostically decisive criteria for tumorous liver lesions can be better identified and whether quantification is possible. Various perfusion analysis methods have been developed for CEUS, such as time intensity curve (TIC) analysis [2,16,17,18]. Until now, the analysis of vascular patterns and vessel density has been of secondary importance for characterization. However, in terms of image morphology, this is crucial for assessing neovascularization and the potential response to therapy.

The aim of this pilot study was to use the novel SR CEUS technique to detect liver lesions and characterize them in terms microvascularization changes using new methods to quantify microvascular patterns.

## 2. Materials and Methods

### 2.1. Ethical Statement

This study was approved by the local ethics committee of the University of Regensburg (Nr. 24-3773-104).

In addition, the university’s interdisciplinary ultrasound center, where this study was conducted, is a reference center for German Society for Ultrasound in Medicine (DEGUM) studies [9] with comprehensive ethical approvals, including prospective randomized studies, and a reference center for CEUS evaluation.

### 2.2. Technical Aspects

The detection of microvascular structures or the detailed microcirculation in organs is difficult using ultrasound imaging modalities. On the other hand, SR CEUS, a modern technology based on optical ultrasound localization microscopy, achieves high-spatial-resolution microbubble localization to overcome these challenges [19,20,21]. This novel imaging modality compromises the conflict between penetration-depth and resolution-quality. SR CEUS achieves high resolution (500 frames per second to acquire images), providing three different imaging maps (vascular density direction, velocity, and velocity direction). Because high-frame-rate (HiFR) CEUS provides individual localization and tracking of the injected microbubbles and their movement, a defined map of the density or velocity of these microbubbles can be produced [22]. SR CEUS is performed through pattern fluctuations of contrast agents within a very short time (*t* ≈ 3 s), allowing better visualization of the microvasculature in different organs, especially in the liver [3].

SR CEUS processing requires DICOM raw data. These data are dependent on devices and cannot be transmitted regularly as data records. We performed post-processing using machine-integrated software. Currently, only CEUS perfusion analysis is independently possible using external software, Vuebox^®^ (Bracco) (https://www.bracco.com/en-ch/product/vuebox (accessed on 15 June 2025)) [23].

### 2.3. Ultrasound Performance

An experienced examiner (>3.000 examinations/year, >20 years) performed all examinations using a convex probe (SC7-1U, 1–7 MHz frequency range) and a high-end ultrasound device (Resona A20, Mindray, Shenzhen, China). Written informed consent was obtained in all cases. The CEUS liver diagnostic tests complied with the European Federation of Societies for Ultrasound in Medicine and Biology (EFSUMB) guidelines for on-label use.

In the first step, we measured the liver area using B-mode ultrasound, color-coded Doppler sonography (CCDS), and ultramicro angiography (UMA). Documentation was performed using short cine loops and single frames. When possible, we also assessed tumor lesions with parameters adapted to low flow (low scale, low PRF, high color gain and low wall filter less than 100 kHz). In the next step, we applied HiFR CEUS after a single injection of 1 to 2.4 mL of SonoVue^®^ (Bracco, Milan, Italy, contrast agent). We performed SR CEUS using a 5-second s cine loop of HiFR CEUS images with a maximum frame rate of 500 frames per second. In the density map of the SR CEUS, the vascular distribution was color-coded from dark red to white (dark red = minimum, red = low, orange = midfield, yellow = high, white = maximum) and compared with the original CEUS images. The measurements of the vascular diameter were implemented in four defined regions of interests (ROIs) with structure-based lines, designed in the centers and margins of the ROI (1 = hilar vessels region; 2 = intrahepatic lesion from the center to the edge; 3 = inconspicuous liver parenchyma region; 4 = subcapsular vascular region).

In general, the arterial, portal, and venous system are distinguished in the blood circulation of the liver. In addition, a distinction is made between the lesion hilum area, lesion central area, liver parenchyma, and capsule region. In this study, our evaluation focuses on microvascularization. This condition affects parts of the parenchyma and the parenchyma area close to the capsule (end stream to capillary level). We always assessed the macrostream pathways regarding *A. hepatica*, portal vein, and hepatic veins hemodynamically using CCDS. The spectral analysis was routinely described as mono-, bi- or triphasic. In *A. hepatica*, we determined the resistance index (RI) extrahepatically and, if necessary, also intrahepatically on different sides in cases of reduced perfusions.

In cases of several liver lesions, we selected the best circumscribable lesion. We measured the capillary density and vessel diameter around these structure-based lines. The results were automatically color-coded, and the envelope curves were presented and transferred into a statistical tabular program.

In ablative procedures, a tumor board decision was first made. Subsequently, a robotic-assisted microwave ablation procedure with a curative approach was performed for malignant liver lesions. Reference imaging and follow-up were performed using magnetic resonance imaging (MRI) with liver-specific contrast agents over a period of 6 months. Treatment was considered successful when there was no vascularization in the ablation area.

### 2.4. Ultrasound Contrast Agent Performance

For dynamic CEUS, a bolus of 1 to 2.4 mL of sulfur-hexafluoride microbubbles (SonoVue^®^, Bracco, Milan, Italy) with a subsequent intravenous bolus administration of 10 mL of saline solution was injected. Low renal function or alterations of thyroid function were not contraindications for injection according to the EFSUMB guidelines [7]. Examinations were achieved in the used picture archiving and communication system (PACS).

### 2.5. Readings

We performed an analysis of image quality and quantification of perfusion, with grades ranging from 1 to 5 (0 = not representative, 1 = partially representative, 2 = representative but with relevant limitations, 3 = adequate, 4 = good, 5 = excellent). Two experienced ultrasound examiners evaluated the images to ensure that the material had sufficient image quality. In addition, the data were collected, documented, and stored in accordance with the applicable data protection guidelines.

### 2.6. Statistical Analysis

All variables were summarized using absolute and relative frequencies, mean ± standard deviation, and median with minimum and maximum values as appropriate for the whole cohort and the four groups. Because of the exploratory nature of this study, no power calculation was performed.

We fitted ordinal regression models with ratings as dependent variables and visualized the results using estimated marginal means. In addition, we applied the nonparametric studentized permutation test from Neubert and Brunner [24] to compare different groups. For the pairwise comparison of different liver regions, we used the sign test. Furthermore, for all regions but the lesion, we fitted linear regression models with the measurements as the dependent variable and the group as well as the age as independent variables. For the lesion, we first modeled the probability of measuring the value zero by applying a logistic regression model and visualized the results using estimated marginal means. Second, we fitted a linear regression model with the log-transformed measurement as the dependent variable using only non-zero values. For the subgroup analysis of benign cases, we proceeded analogously; however, to address complete separation, we applied Firth’s correction for logistic regression.

The significance level was set to *p* < 0.05 for all statistical tests. To account for multiple testing, we adjusted *p*-values using the Holm–Bonferroni method. Results were interpreted based on these adjusted *p*-values.

We performed all analyses using the statistical programming environment R (version 4.2.3; [25]) using the R packages ggstatsplot (version 0.12.4; [26]) for box–violin plots, BSDA (version 1.2.2; [27]) for the sign test, ordinal (version 2023.12.4.1; [28]) for ordinal regression models, ggeffects (version 1.7.1; [29]) for the computation and visualization of estimated marginal means and logistf (version 1.26.1; [30]) for logistic regression with Firth’s correction.

## 3. Results

We evaluated the data from 65 patients (*n* = 28 female; *n* = 37 male; average age 57.8 ± 17.2 years) (see Table 1). The examined lesions in the liver were mostly benign tumors (*n* = 46). Most patients had mild liver fibrosis (stage F1) (*n* = 35). The measurements were performed using the shear-wave technique in accordance with the guidelines [31] in respiratory arrest, not on the capsule, at a depth of 2 cm to a maximum of 5 cm, with at least six measurements considered and a maximum deviation of 5% in the measurement. The reference values correspond to those in the guidelines, for F1 < 1.8 m/s / < 9 kPa.

**Figure 1:**
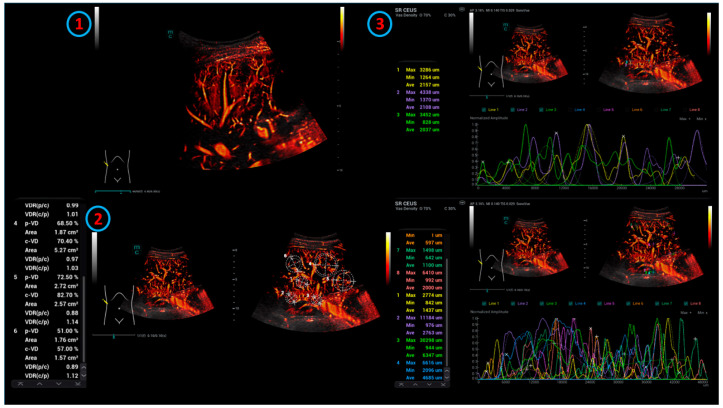
Examination of the liver using SR CEUS: 1. Imaging of liver vessels in the intercostal section using SR CEUS technology. 2. Determination of ROIs. 3. ROIs are surveyed using structure-based lines.

The lesions examined (Figure 1) were at a mean tissue depth of 6.07 cm, starting from the probe lying on the skin.

**Figure 2:**
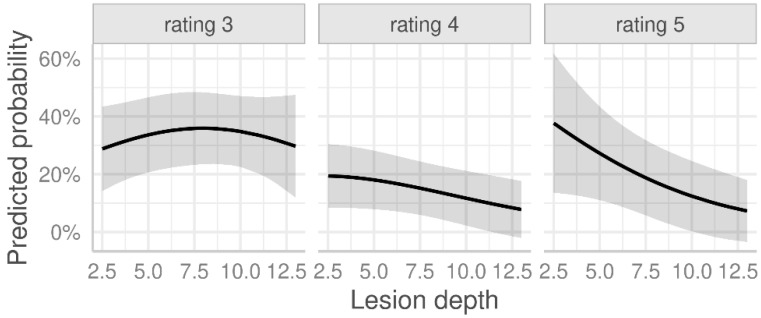
Predicted probabilities of SR CEUS ratings 3 to 5 (adequate, good and excellent, respectively) depending on the lesion depth (cm), defined as the distance from the skin to the center of the lesion, as estimated from an ordinal regression model (see Table A1 in the Appendix A). The model’s predictions are accompanied by 95% confidence bands (shaded area).

A closer analysis of the influence of lesion depth on image quality and the ability to evaluate SR CEUS revealed that quality of the image deteriorated as the tissue depth increased. An increasing tissue depth is significantly associated with lower image quality in the microcirculation evaluation using SR CEUS (see Figure 2).

**Figure 3:**
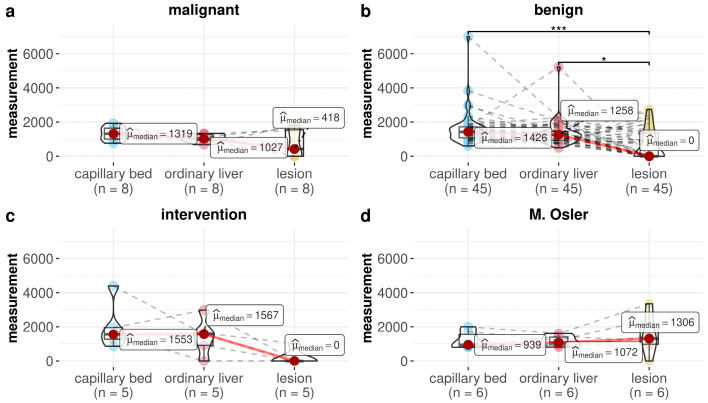
Box–violin plots comparing the different liver regions for malignant (**a**) and benign (**b**) cases, the intervention group (**c**), and patients with Morbus Osler (**d**). For a complete list of *p*-values, see Table A2 in the Appendix A. * *p* < 0.05, *** *p* < 0.001.

**Figure 4:**
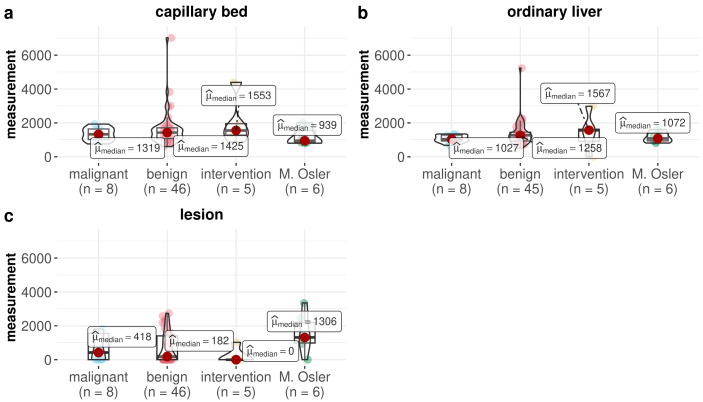
Box–violin plots comparing the measurements in the capillary bed (**a**), ordinary liver (**b**), and lesion (**c**) between the four groups. No significant differences were detected between the groups (see Table A3, Table A4 and Table A5 in the Appendix A).

Comparisons of the measured microvascular parameters revealed differences between the entities and examined liver regions (see Figure 3 and Figure 4).

**Figure 5:**
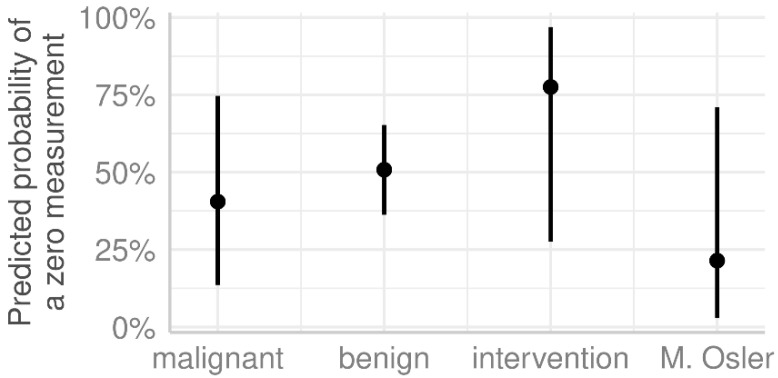
The predicted probability of zero measurement for the lesion in the four groups with 95% confidence intervals. The probabilities were estimated using a logistic regression model and are adjusted for age (see Table A4 in the Appendix A).

In the malignant lesions, we obtained lower measurements in the lesion itself than in normal liver tissue or the hepatic capillary region. This pattern was also repeated for benign lesions. We observed significant differences in the measurements in the benign group in the capillary region against lesion (*p* < 0.001) and normal liver tissue against the lesion (*p* = 0.002). In contrast, the Morbus Osler group showed the exact opposite picture, with the highest number of measurements in the lesions. However, these measurements were not significantly different from those in the capillary region (*p* = 0.219) or normal liver tissue (*p* = 1.000). Further differentiation based on the examined liver regions revealed that the highest values for the lesion were found in the malignant lesion group and Morbus Osler group, although no significant differences were detected (see Table A4 and Table A5 in the Appendix A). Additional analysis showed that the calculated probability of an undetectable measurement was highest in the intervention group and lowest in the Morbus Osler group (Figure 5).

Only the benign group was further subdivided, where possible, because in the other main groups, the corresponding subgroups would be too small for meaningful statistical evaluation due to the sample size available. In this subgroup analysis, we analyzed FNH (*n* = 6), hemangioma (*n* = 13), and cyst (*n* = 11) as subgroups of benign cases (see Table A6 in the Appendix A). We observed no significant differences between these subgroups for the capillary bed or ordinary liver (see Table A7 in the Appendix A). However, undetectable measurements for lesions occurred significantly more frequently in the cyst subgroup compared with the FNH subgroup (see Table A8 in the Appendix A). When considering only detectable measurements, FNH had significantly higher values than hemangioma did (see Table A9 in the Appendix A).

## 4. Discussion

Modern ultrasound techniques enable increasingly accurate imaging and characterization of tumor manifestations.

Using modern SR CEUS technology, the detection and characterization in vascularization mode can be achieved by displaying the vascularization patterns of the liver and liver lesions or other organs, which was previously possible only with superselective catheter angiography [3,32]. For a final characterization, the wash-in and wash-out effects of the lesions to be evaluated must always be considered [7,12]. This is where the SR CEUS technique offers an advantage, with adequate tissue penetration and high detail accuracy combined with higher frame rates. The image corresponds to that obtained by digital subtraction angiography. Zone sonography generates a broadband sound wave, thereby destroying less of the contrast agent and making it detectable for longer periods of time. Arterial blood flow can be visualized in various phases down to the capsule level and subsegmental arterioles. In later phases, portal venous flow also becomes visible. This is particularly the case when shunt connections—such as those found in tumors—cause premature contrast enhancement of the venous system. The advantage of this technique lies in the visualization of arterial blood flow without superimposition. This allows vascular patterns to be better differentiated as regular (benign) or irregular (malignant).

Image quality correlates with penetration depth, with probability models showing sufficient quality for liver assessment. However, deeper structures, especially those located 7.5 cm or deeper, may already show relevant limitations for an adequate evaluation of ultrasound images in more than 30% of the cases. At present, all CEUS examinations are dependent on the surrounding structures, particularly in cases of high body mass index and previous attenuation up to the liver capsule of up to 5 cm, which can lead to loss of image quality. Similarly, steatosis and cirrhosis—depending on their severity—as well as colon interposition and air overlays, alter the acoustic conditions and thus the quality of the ultrasound. Nevertheless, it was demonstrated that even under limited conditions, liver lesions can be detected and characterized with high diagnostic accuracy (>85%) at a penetration depth of up to 15 cm [33].

In the evaluation of benign lesions, SR CEUS can help clarify the regular vascularization pattern of solid FNH lesions. In accordance with the guidelines for benign lesions based on multicenter studies [9] for FNH, adenoma, and hemangioma are follow-up examinations over at least one year and reference imaging using MRI with liver-specific contrast agents available. Differentiation from an adenoma may also be considered difficult in examinations using MRI with liver-specific contrast agents [34] if slight wash-out can be detected in CEUS. Hemangioma assessment can also be more complex or overlap with intrahepatic metastases [35,36]. In partially thrombosed hemangiomas, which often have little echo, part of the contrast is missing in the center in the late phase, which can resemble necrosis. This then leads to specific questions requiring further clarification after contrast-enhanced computed tomography (CT) with CEUS and, if available, SR CEUS, which was also significantly demonstrated in the subgroup analysis we performed. The supplementary measurement tools also improved the delineation of cysts and complicated cysts. We monitored cysts over a period of at least 2 years and classified them as benign based on echo enhancement, marginal shadows without relevant internal changes, and septations smaller than 2 mm [37].

We observed a pronounced degree of neovascularization or vascular changes could be seen in the lesions of both the malignant and Morbus Osler group. The measured values showed particularly high values for this. All malignant lesions were histologically confirmed during follow-up examinations after the tumor board decisions were made, as part of surgical or ablative procedures. However, we used SR CEUS to assess the vascular pattern but not to determine the extent of the lesions. It is known from the literature that the measurable diameter of malignant lesions changes dynamically from the early arterial phase to the late wash-out phase and increases in size. However, it remains the same in benign lesions [7]. In a highly specialized university center for vascular malformations, Morbus Osler is a relevant entity for the exclusion of vascular tumors or shunts using CEUS. We used the new SR CEUS technique to examine the extent to which tumor-like forms of microshunts can be imaged. These findings constitute the prerequisite for the use of SR CEUS in relation to the detection of dynamic tumor neovascularization. Therefore, we examined a separate group, Morbus Osler, for the initial orientation assessment.

However, possibly because of the small group sizes, no significant differences were detected when compared to the benign and intervention groups. In smaller tumor foci, the extent of irregular neovascularization in particular may be the main criterion for the detection of HCC tumor foci according to currently valid criteria [7,8,9,38,39]. In lesions with a maximum diameter of 10 mm, wash-out may be detectable to only a limited extent, and it may even be absent in the late phase. In the case of cholangiocarcinoma (CCC) metastases, wash-out is detectable earlier and more effectively than that in HCC. It should be noted here that ultrasound contrast agents, such as SonoVue^®^, are vascular and therefore not directly comparable with liver-specific contrast agents [40]. This further emphasizes the importance of SR CEUS through improved dynamic imaging of microvascularization down to the capillary level.

After ablation, chemoembolization (TACE), and especially postoperatively, the goal should be an avascular defect or unremarkable scar [41]. This should mean that similar to an uncomplicated cyst, no microvascularization or marginal irregular nodular hyperemia should be detectable in the SR CEUS. This was also demonstrated in the intervention group studied. Nevertheless, particularly after TACE, only partial devascularization can occasionally be achieved. The quantification tools of SR CEUS are well suited for corresponding assessments, although corresponding scientific analyses are still pending. Other assessment techniques include evaluation with a perfusion tool, such as Time Intensity Curve analysis (TIC) [2], or parametric display with false color analysis [42]. Whether the elevated values are due to residual tumor tissue in the surrounding area or new tumor lesions can be adequately assessed only by follow-up examinations and reference imaging, such as contrast-enhanced CT or MRI.

In addition to the malignant, benign, and postinterventional groups examined herein, cysts and cystic tumors play a relevant role in daily clinical practice [43]. The distinction between cysts, complicated cysts, and reactive inflammatory cysts can also be made on SR CEUS, similar to the Bosniak criteria for renal cysts, based on the thickness of possible septa and regular or irregular tumorous nodular contrast enhancement with wash-out. In simple cysts, SR CEUS is not able to show vascularization as a diagnostic criterion. However, inflammation with melting may lead to hyperemia with increased contrast at the margins, which persists into the late phase and can be detected with a high degree of probability using the SR CEUS technique. Abscesses also contain air pockets in some areas. Reference images obtained by CT were available for abscesses that were punctured percutaneously and, where possible, treated with drainage.

Cystic tumors are irregular, have membranes < 3 mm, and may contain nodular solid components that are arterially hypervascularized and exhibit wash-out in the tumor area in the late phase. In these cases, which can sometimes be difficult to differentiate, the original dynamic SR CEUS images should also be evaluated, if available. In some cases, further differentiation can be achieved using the measurement tools. The additional use of perfusion analysis may be helpful here, including parametric imaging with false color analysis [2,42]. Although other techniques, such as elastography, can also be used, it is important to note that artifact superimposition can occur in cysts and complicated cysts. SR CEUS has the potential to complement MRI in providing clearer differentiation of tumor cysts and complicated cysts, particularly in cases in which clear assessment is difficult.

In addition to appropriate measurement tools, the use of high-resolution probe technology plays an important role in the proper assessment of lesions. The multi-frequency range (1–7 MHz) offers good depth penetration and detail accuracy in the near field.

In the present study design, a TIC analysis was not possible within a sufficient time frame. At this time, raw CEUS data cannot be stored for SR CEUS. The recording of CEUS was interrupted during the early arterial, arterial, and late arterial phases, when SR CEUS examinations were performed. Because the early onset phase was a maximum of 15 s, it was not possible to perform an adequate TIC analysis (e.g., evaluation of time to peak or area under the curve (AUC)) as the maximum contrast was not reached. However, studies have shown that CEUS perfusion can make a decisive contribution to better characterizing and differentiating tumor entities, as well as assessing the success of treatment [38].

Overall, the advantage of vascularization imaging using SR CEUS lies in its clear and detailed visualization of liver vascularization in the arterial and portal venous phases, as well as tumor neovascularization. In view of the correlation between microvessel density and the progression of certain diseases (e.g., tumors or kidney disease), increasingly detailed imaging of these vascular architectures plays an important role [44,45]. However, these vivid vessel reconstructions have limitations, such as susceptibility to pulsation and potential artifact superposition. Lesions in close proximity to the heart, especially segment II/III, are often obscured by artifacts. Such limitations must be considered when using this technique [32]. Sufficient practical experience of the respective ultrasound examiners in handling SR CEUS is therefore essential for the optimal application of this modern technology. The main problems are the artifacts known from sound technology: reflection, attenuation, and dispersion. In general, the surrounding tissue influences liver examination. Thus, sound changes are also expected for CEUS in the interface area to the diaphragm, pleura, gallbladder, and adjacent intestinal loops due to reflection, mirroring, or attenuation [7]. Improvement in examination conditions can influence possible artifacts. If patients are examined on an empty stomach, prepared for ultrasound examination, and positioned optimally, the penetration depth and ultrasound conditions can be optimized. In addition, adjusting the amount of contrast agent can contribute to an improvement. In more difficult ultrasound conditions, the dose of contrast agent is usually increased from 1.5 to 2.4 mL Sonovue^®^ as a bolus. In addition, the CEUS mode allows switching from pulse-inversion mode to amplitude amplification with greater penetration depth.

Among other things, SR CEUS will be tasked with the detailed vascular imaging for the dynamic analysis of wash-in and wash-out kinetics, another important aspect of multimodal imaging of tumor vascularization.

To gain a better understanding of the capabilities and clinical application of this advanced technology, further studies, including large-scale prospective multicenter studies, are needed. Additional observations should also be analyzed regarding malignant tumors originating in the liver, such as HCC or CCC, in comparison with metastases.

## 5. Limitations

This is an exploratory retrospective analysis of a small cohort of patients. We did not perform further differentiation according to specific tumor entities because this would have resulted in comparison groups that were too small. Comparisons between individual tumor entities in larger patient cohorts may lead to new or different findings. It should also be noted that, with the exception of the benign tumor group, only negligible group sizes were available. In addition, we excluded previous systemic therapies and any associated effects on the vascular architecture from the evaluation. In addition, future studies should include independent evaluations by several investigators and analyze interobserver consistency using statistical testing.

## Figures and Tables

**Table 1:** Demographic and measurement data from the patient cohort studied.

	Malignant (*n* = 8)	Benign (*n* = 46)	Intervention (*n* = 5)	Morbus Osler (*n* = 6)	Overall (*n* = 65)
**Age, years**					
Mean (SD)	70.6 (10.2)	56.7 (17.6)	63.2 (7.82)	45.0 (17.3)	57.8 (17.2)
Median [min, max]	73.5 [52.0, 84.0]	57.0 [22.0, 88.0]	59.0 [55.0, 73.0]	47.0 [22.0, 64.0]	59.0 [22.0, 88.0]
**Sex**					
Male	6 (75.0%)	23 (50.0%)	5 (100%)	3 (50.0%)	37 (56.9%)
Female	2 (25.0%)	23 (50.0%)	0 (0%)	3 (50.0%)	28 (43.1%)
**Steatosis**					
No steatosis	6 (75.0%)	17 (37.0%)	3 (60.0%)	4 (66.7%)	30 (46.2%)
Steatosis	2 (25.0%)	29 (63.0%)	2 (40.0%)	2 (33.3%)	35 (53.8%)
**Fibrosis**					
F0	1 (12.5%)	12 (26.1%)	0 (0%)	1 (16.7%)	14 (21.5%)
F1 fibrosis	5 (62.5%)	24 (52.2%)	2 (40.0%)	4 (66.7%)	35 (53.8%)
F2 fibrosis	0 (0%)	6 (13.0%)	2 (40.0%)	0 (0%)	8 (12.3%)
F3 fibrosis	1 (12.5%)	2 (4.3%)	0 (0%)	1 (16.7%)	4 (6.2%)
F4 fibrosis	1 (12.5%)	2 (4.3%)	1 (20.0%)	0 (0%)	4 (6.2%)
**Lesion depth** **(center), cm**					
Mean (SD)	5.75 (2.13)	5.87 (2.35)	8.66 (2.56)	5.83 (3.07)	6.07 (2.47)
Median [min, max]	5.00 [3.40, 9.90]	5.25 [2.50, 13.0]	8.20 [5.10, 12.0]	4.25 [3.20, 10.0]	5.10 [2.50, 13.0]
**Lesion height,** **cm**					
Mean (SD)	2.87 (1.89)	2.64 (1.73)	3.01 (0.816)	3.98 (1.77)	2.80 (1.71)
Median [min, max]	1.91 [1.36, 7.00]	2.36 [0.57, 7.40]	2.88 [2.00, 3.93]	4.00 [1.50, 6.51]	2.50 [0.57, 7.40]
Missing	0 (0%)	0 (0%)	0 (0%)	1 (16.7%)	1 (1.5%)
**Lesion width,** **cm**					
Mean (SD)	2.64 (1.72)	2.48 (1.58)	2.80 (0.716)	2.35 (0.899)	2.51 (1.48)
Median [min, max]	1.93 [1.15, 6.00]	2.02 [0.54, 7.07]	2.50 [2.00, 3.80]	2.32 [1.50, 3.61]	2.25 [0.54, 7.07]
Missing	0 (0%)	0 (0%)	0 (0%)	1 (16.7%)	1 (1.5%)

## Data Availability

All relevant data are contained within the paper. The transcripts analyzed in this study are available from the corresponding author upon reasonable request.

## Abbreviations

The following abbreviations were used in this manuscript:

SR CEUS	Super-resolution contrast-enhanced ultrasound
CEUS	Contrast-enhanced ultrasound
FNH	Focal nodular hyperplasia
HCC	Hepatocellular carcinoma
RI	Resistance index
DEGUM	German Society for Ultrasound in Medicine
TACE	Transarterial embolization
TIC	Time intensity curve
HiFR	High frame rate
CCDS	Color Doppler sonography
UMA	Ultra-micro-angiography
ROI	Regions of interests
PACS	Picture archiving and communication system
MRI	Magnetic resonance imaging
AUC	Area under the curve
CT	Computed tomography
CCC	Cholangiocarcinoma

## Appendix A

**Table A1:** Multivariable ordinal regression models for the quality ratings for SR CEUS.

Variable	OR	95% CI	*p*-Value
**Age**	0.98	(0.95, 1.01)	0.123
**Lesion depth (cm)**	0.82	(0.67, 1.00)	*0.047*
**Fibrosis**			
F0 + F1	1.00	Ref.	
F2	1.73	(0.46, 6.70)	0.420
F3 + F4	2.54	(0.52, 13.1)	0.251
**Steatosis**			
No steatosis	1.00	Ref.	
Steatosis	0.55	(0.20, 1.50)	0.245

OR = Odds Ratio; CI = confidence interval.

**Table A2:** Pairwise comparison of liver regions using sign tests. The *p*-values were adjusted for multiple testing using the Holm–Bonferroni method.

Group	Pairwise Comparison	Unadjusted *p*-Value	Adjusted *p*-Value
Malignant	Ordinary liver vs. lesion	0.727	1.000
Malignant	Capillary bed vs. lesion	0.070	0.630
Malignant	Capillary bed vs. ordinary liver	0.289	1.000
Benign	Ordinary liver vs. lesion	*0.002*	*0.022*
Benign	Capillary bed vs. lesion	*<0.001*	*<0.001*
Benign	Capillary bed vs. ordinary liver	0.072	0.630
Intervention	Ordinary liver vs. lesion	0.125	0.875
Intervention	Capillary bed vs. lesion	0.063	0.630
Intervention	Capillary bed vs. ordinary liver	1.000	1.000
M. Osler	Ordinary liver vs. lesion	1.000	1.000
M. Osler	Capillary bed vs. lesion	0.219	1.000
M. Osler	Capillary bed vs. ordinary liver	1.000	1.000

**Table A3:** Multivariable linear regression models for capillary bed and ordinary liver. The *p*-values were adjusted for multiple testing using the Holm–Bonferroni method.

	Capillary Bed				Ordinary Liver			
Variable	N	β	95% CI	Adjusted *p*-Value	N	β	95% CI	Adjusted *p*-Value
**Group**				0.870				0.870
Malignant	8	0	Ref.		8	0	Ref.	
Benign	46	501	(−248, 1250)	0.372	45	293	(−271, 858)	0.372
Intervention	5	798	(−279, 1876)	0.288	5	347	(−466, 1160)	0.397
M. Osler	6	294	(−789, 1377)	1.000	6	64	(−755, 883)	1.000
**Age**	65	16	(1, 31)	0.064	65	−1	(−13, 10)	0.825

β = regression coefficient; CI = confidence interval.

**Table A4:** Multivariable logistic regression for the probability of obtaining a zero measurement for the lesion.

Variable	N	No. of Events	OR	95% CI	*p*-Value
**Group**					0.323
Benign	46	23	1.00	Ref.	
Malignant	8	4	0.66	(0.13, 3.33)	0.608
Intervention	5	4	3.34	(0.44, 68.7)	0.301
M. Osler	6	1	0.26	(0.01, 1.94)	0.250
**Age**	65	32	1.03	(1.00, 1.07)	0.080

OR = Odds Ratio; CI = confidence interval.

**Table A5:** Multivariable linear regression model for the non-zero measurements for the lesion. The dependent variable (i.e., the non-zero measurements) was log-transformed.

Variable	N	Exp(β)	95% CI	*p*-Value
**Group**				0.691
Malignant	4	1.00	Ref.	
Benign	23	0.94	(0.51, 1.70)	0.820
Intervention	1	0.73	(0.23, 2.35)	0.591
M. Osler	5	1.23	(0.57, 2.67)	0.587
**Age**	33	1.00	(0.99, 1.01)	0.824

exp(β) = ratio of means; CI = confidence interval.

**Table A6:** Descriptive values for the subgroups FNH, cyst and hemangioma.

	FNH (*n* = 6)	Hemangioma (*n* = 13)	Cyst (*n* = 11)
**Measurement for capillary bed**			
Median [Min, Max]	1136 [599, 1881]	1424 [1078, 3831]	1530 [960, 2133]
**Measurement for ordinary liver**			
Median [Min, Max]	919 [493, 1841]	1223 [723, 2135]	1282 [704, 2251]
Missing	1 (16.7%)	0 (0%)	0 (0%)
**Measurement for lesion**			
Median [Min, Max]	2235 [1042, 4716]	1308 [0, 7622]	0 [0, 1200]

**Table A7:** Multivariable linear regression models for capillary bed and ordinary liver, considering benign cases only. The *p*-values were adjusted for multiple testing using the Holm–Bonferroni method.

	Capillary Bed				Ordinary Liver			
Variable	N	β	95% CI	Adjusted *p*-Value	N	β	95% CI	Adjusted *p*-Value
**Group**				0.890				0.993
FNH	6	0	Ref.		6	0	Ref.	
Hemangioma	13	165	(−534, 864)	1.000	13	−22	(−603, 559)	1.000
Cyst	11	−147	(−903, 609)	1.000	11	−35	(−659, 588)	1.000
**Age**	30	17	(1, 33)	0.072	29	11	(−1, 24)	0.081

β = regression coefficient; CI = confidence interval.

**Table A8:** Multivariable logistic regression with Firth correction for the probability of obtaining a zero measurement for the lesion.

Variable	N	No. of Events	OR	95% CI	*p*-Value
**Group**					*0.012*
FNH	6	0	1.00	Ref.	
Hemangioma	13	4	2.26	(0.07, 383)	0.646
Cyst	11	10	30.6	(1.35, 5.594)	*0.030*
**Age**	30	14	1.04	(0.98, 1.14)	0.188

OR = Odds Ratio; CI = confidence interval.

**Table A9:** Multivariable linear regression model for the non-zero measurements for the lesion. The dependent variable (i.e., the non-zero measurements) was log-transformed. The subgroup cyst was excluded in this study because it had only one non-zero measurement.

Variable	N	Exp(β)	95% CI	*p*-Value
**Group**				0.004
FNH	6	1.00	Ref.	
Hemangioma	9	0.43	(0.26, 0.73)	0.004
**Age**	15	1.03	(1.01, 1.05)	0.824

exp(β) = ratio of means; CI = confidence interval.
